# Postoperative systemic inflammatory response syndrome predicts increased mortality in patients after elective craniotomy

**DOI:** 10.3389/fsurg.2023.1331073

**Published:** 2024-01-04

**Authors:** Liyuan Peng, Qi Gan, Yangchun Xiao, Jialing He, Xin Cheng, Peng Wang, Lvlin Chen, Tiangui Li, Yan He, Weelic Chong, Yang Hai, Chao You, Fang Fang, Yu Zhang

**Affiliations:** ^1^ Department of Critical Care Medicine, Affiliated Hospital of Chengdu University, Chengdu, Sichuan, China; ^2^ Department of Neurosurgery, West China Hospital, Sichuan University, Chengdu, Sichuan, China; ^3^ Department of Neurosurgery, Affiliated Hospital of Chengdu University, Chengdu, Sichuan, China; ^4^ Department of Medical Oncology, Thomas Jefferson University, Philadelphia, PA, United States; ^5^ Department of Radiology, Thomas Jefferson University, Philadelphia, PA, United States; ^6^ Center for Evidence Based Medical, Affiliated Hospital of Chengdu University, Chengdu, Sichuan, China

**Keywords:** craniotomy, mortality, postoperative, systemic inflammatory response syndrome, prognosis

## Abstract

**Introduction:**

Patients undergoing craniotomy are at high risk of perioperative morbidity and mortality due to excessive inflammatory responses. The purpose of the present study is to evaluate the prognostic utility of postoperative systemic inflammatory response syndrome (SIRS) in patients undergoing craniotomy.

**Methods:**

We performed a retrospective cohort study of patients who underwent craniotomy between January 2011 and March 2021. SIRS was diagnosed based on two or more criteria (hypo-/hyperthermia, tachypnea, leukopenia/leukocytosis, tachycardia). We used univariate and multivariate analysis for the development of SIRS with postoperative 30-day mortality.

**Results:**

Of 12,887 patients who underwent craniotomy, more than half of the patients (*n* = 6,725; 52.2%) developed SIRS within the first 7 days after surgery, and 157 (1.22%) patients died within 30 days after surgery. In multivariable analyses, SIRS (OR, 1.57; 95% CI, 1.12–2.21) was associated with 30-day mortality. Early SIRS was not predictive of 30-day mortality, whereas delayed SIRS was predictive of 30-day mortality. Abnormal white blood cell (WBC) counts contributed the most to the SIRS score, followed by abnormal body temperature, respiratory rate, and heart rate.

**Conclusion:**

Postoperative SIRS commonly occurs after craniotomy and is an independent predictor of postoperative 30-day mortality. This association was seen only in delayed SIRS but not early SIRS. Moreover, increased WBC counts contributed the most to the SIRS score.

## Introduction

Craniotomy is a high-cost surgical procedure that is the standard of care in the treatment of brain tumor, epilepsy, aneurysm, hemorrhagic stroke, and traumatic brain injury. Patients undergoing craniotomy are at a high risk of perioperative morbidity and mortality (1). Indeed, the inflammatory response evoked is a crucial component of surgery. Because this response is necessary to restore normal physiology, an excessive response can cause secondary brain damage and systemic inflammatory response syndrome (SIRS) (1).

SIRS can generate neuroinflammation in the brain and cause tissue damage in the body, which can precipitate further inflammation and damage in systemic tissues (2–4). SIRS is believed to be a systemic reaction to a stimulus (e.g., trauma and surgery). SIRS has been recognized as a risk factor for poor outcomes in neurologic illnesses (i.e., stroke and traumatic brain injuries) (5–8). Previous studies have suggested that SIRS is common and has a negative impact on patient outcomes after various types of surgeries (9–15). However, the prognostic significance of SIRS in patients undergoing craniotomy remains unclear. The purpose of the present study is to evaluate the prognostic utility of postoperative SIRS in patients undergoing elective craniotomy.

## Methods

### Study design and patient selection

We retrospectively examined adult patients who underwent elective craniotomy. Data were collected from consecutive electronic health records of West China Hospital, Sichuan University between January 2011 and March 2021. Our study was conducted in accordance with the principles announced in the Declaration of Helsinki. The study was approved by the ethics committee of West China Hospital with a waiver of informed consent (approval number: 2022-705; approval date, April 29, 2022; study title, Studies on the risk and prognosis of neurosurgery).

### Patient selection

We included adults (≥18 years old) undergoing elective craniotomy. We excluded (1) patients undergoing repeat resection or burr hole surgery; (2) patients who underwent emergency or urgent craniotomy; (3) patients with infections during the entire hospital stay; and (4) patients whose identity card number was not available or whose death record was not found in The Chinese Hukou System (referred to as Household Registration Administration System (16). Chinese law stipulates that if a citizen dies, the dependent, head of household, relative, or community should report the death registration and cancel the residence registration of the person within 30 days. Hence, the system has the most accurate message about death.

### SIRS

The main exposure was postoperative SIRS in the 7 days after surgery. SIRS was determined by abnormalities in white blood cell (WBC) counts, heart rate, respiratory rate, and body temperature and was defined according to international convention when two or more of the following criteria were present (17):

1. Body temperature > 38°C or body temperature < 36°C
2. Heart rate > 90 bpm
3. Respiratory rate >20 bpm or PaCO2 < 32 mmHg
4. WBC count >12,000 cells/mm^3^ or <4,000 cells/mm^3^ or the presence of >10% immature neutrophils (bands)

Location- and time-stamped vital signs (respiratory rate, heart rate, and body temperature) were collected from consecutive electronic health records. The PaCO_2_ and immature band criteria were unavailable and were therefore not used in the study. In the case of controlled mechanically ventilated patients, one was assigned as the criterion of respiratory rate. To investigate the SIRS score, we selected the worst vital signs and leukocyte value daily.

Within 7 days after surgery, patients with two or more SIRS criteria were considered “SIRS” positive, whereas those who presented within the first 3 days were considered “early SIRS” and those from days 4 and 7 were considered “delayed SIRS.”

### Other variables

Preoperative data obtained included demographics (including age, gender, alcohol use, and tobacco use), relevant comorbidities (hypertension, diabetes, coronary artery disease, chronic liver disease, current dialysis), intraoperative variables (surgery time, intraoperative blood loss), primary diagnosis, perioperative steroid use, American Society of Anesthesiologists (ASA) class, and other variables (Table 1). If a patient underwent more than one surgery during the hospitalization, only the procedural characteristics of the first surgery were included in the analysis.

**Table 1:** Baseline characteristics of the patients by postoperative SIRS.

Characteristics	No SIRS	SIRS	*p*
	(*n* = 6,162)	(*n* = 6,725)	
Demographics			
Age, year, mean (SD)	49.51 (13.59)	48.07 (13.82)	<0.001*
Female, *n* (%)	3,625 (58.8)	3,659 (54.4)	<0.001*
Smoking, *n* (%)	684 (11.1)	782 (11.6)	0.360
Alcohol, *n* (%)	892 (14.5)	1,074 (16.0)	0.020*
Medical history, *n* (%)			
Hypertension	932 (15.1)	1,112 (16.5)	0.030*
Diabetes	438 (7.1)	380 (5.7)	0.001*
Chronic liver disease	285 (4.6)	265 (3.9)	0.061
Coronary artery disease	57 (0.9)	52 (0.8)	0.399
Current dialysis	82 (1.3)	59 (0.9)	0.017*
Primary diagnosis, *n* (%)			
Benign tumor	2,979 (48.3)	3,398 (50.5)	<0.001*
Malignant tumor	1,125 (18.3)	1,583 (23.5)	
Other	1,348 (21.9)	1,012 (15.0)	
Vascular	710 (11.5)	732 (10.9)	
ASA class, *n* (%)			
I–II	4,001 (64.9)	4,131 (61.4)	<0.001*
III–V	2,161 (35.1)	2,594 (38.6)	
Steroid use, yes, *n* (%)	4,223 (68.5)	5,261 (78.2)	<0.001*
Surgery time, hours	3.51 (1.74)	3.69 (1.79)	<0.001*
Intraoperative blood loss, mL	247.63 (436.33)	285.17 (452.39)	<0.001*
Preoperative biology, mean (SD)			
CRP	11.35 (19.99)	15.96 (26.73)	0.009*
PCT	0.07 (0.29)	0.09 (0.17)	0.431
Neutrophil	5.56 (3.99)	6.77 (4.90)	<0.001*
RDW-CV	13.40 (1.33)	13.40 (1.28)	0.786

SIRS, systemic inflammatory response syndrome; ASA, American Society of Anesthesiologists; CRP, C-reactive protein; PCT, procalcitonin; RDW-CV, coefficient of variation of red blood cell distribution width.

* Means statistically significance.

Inflammatory biomarkers, such as high-sensitive C-reactive protein (CRP), absolute neutrophil count, coefficient of variation of red blood cell distribution width (RDW-CV), and neutrophil–lymphocyte ratio (NLR), were also collected after craniotomy.

### Outcomes

The primary outcome measure was the 30-day postoperative mortality. The date of death was determined from the Chinese Hukou System (16).

### Statistical analysis

Statistical analyses were performed using R software version 4.2.2. A two-sided *p* value less than 0.05 was considered statistically significant. Variables are expressed as mean ± SD or number of patients (percentage). The continuous variables were compared using the Student’s *t*-test or the Mann–Whitney *U*-test. The proportions between groups were compared using the chi-square test.

We used univariate and multiple logistic regression analysis to examine independent risk factors for the development of SIRS positivity, early SIRS, and delayed SIRS with 30-day mortality. Factors influencing outcome with *P* < 0.10 in univariate analysis were implemented into the multivariable analysis. We replaced the missing values with the median for continuous values and others for categorical variables.

Subgroup analyses included age, sex, alcohol use, smoking, diabetes mellitus, hypertension, coronary artery disease, chronic liver disease, steroid use, and ASA class. Bonferroni *p* values < 0.01 were considered statistically significant for the subgroup analyses.

## Results

**Figure 1:**
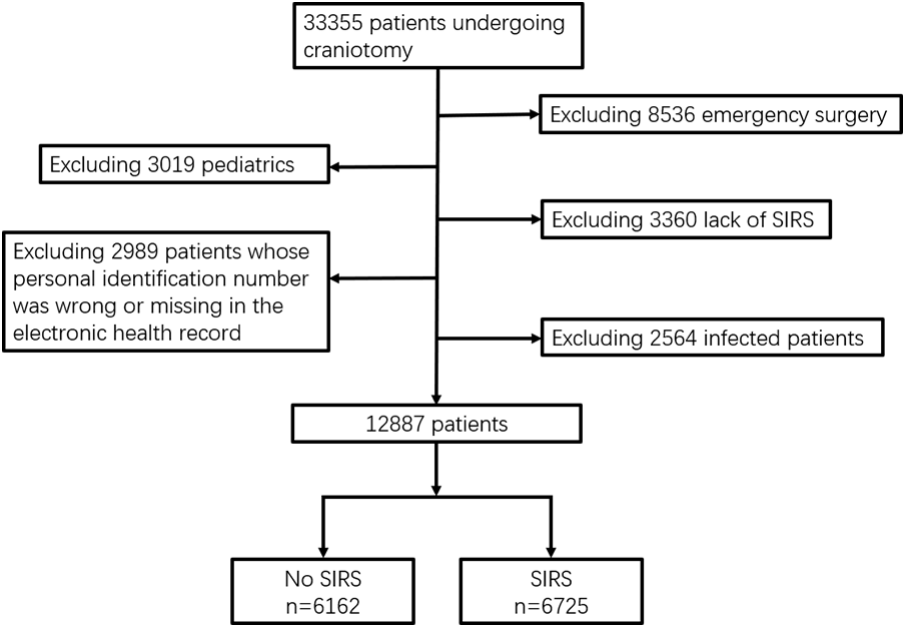
Flowchart.

After 20,468 patients were excluded from the analysis (8,536 patients underwent emergency surgery, 3,019 patients were not adult patients, 3,360 patients missing SIRS-related value, 2,989 patients were missing the death record, and 2,564 patients had infections during the entire hospital stay), 12,887 patients who underwent craniotomy were included in the final analysis (Figure 1).

Of the 12,887 patients who underwent craniotomy, more than half of the patients (*n* = 6,725; 52.2%) developed SIRS within the first 7 days after surgery, and 157 (1.22%) patients died within 30 days after surgery. Baseline characteristics, medical history, intraoperative variables, and preoperative biologics are detailed in Table 1. Postoperative SIRS-positive patients within 7 days were more likely to be younger, male, and have a history of alcohol abuse, hypertension, diabetes, current dialysis, primary diagnosis, high ASA class, perioperative steroid use, long surgery time, intraoperative blood loss, preoperative high CRP, and high neutrophil.

### SIRS criteria over 7 days after craniotomy

**Figure 2:**
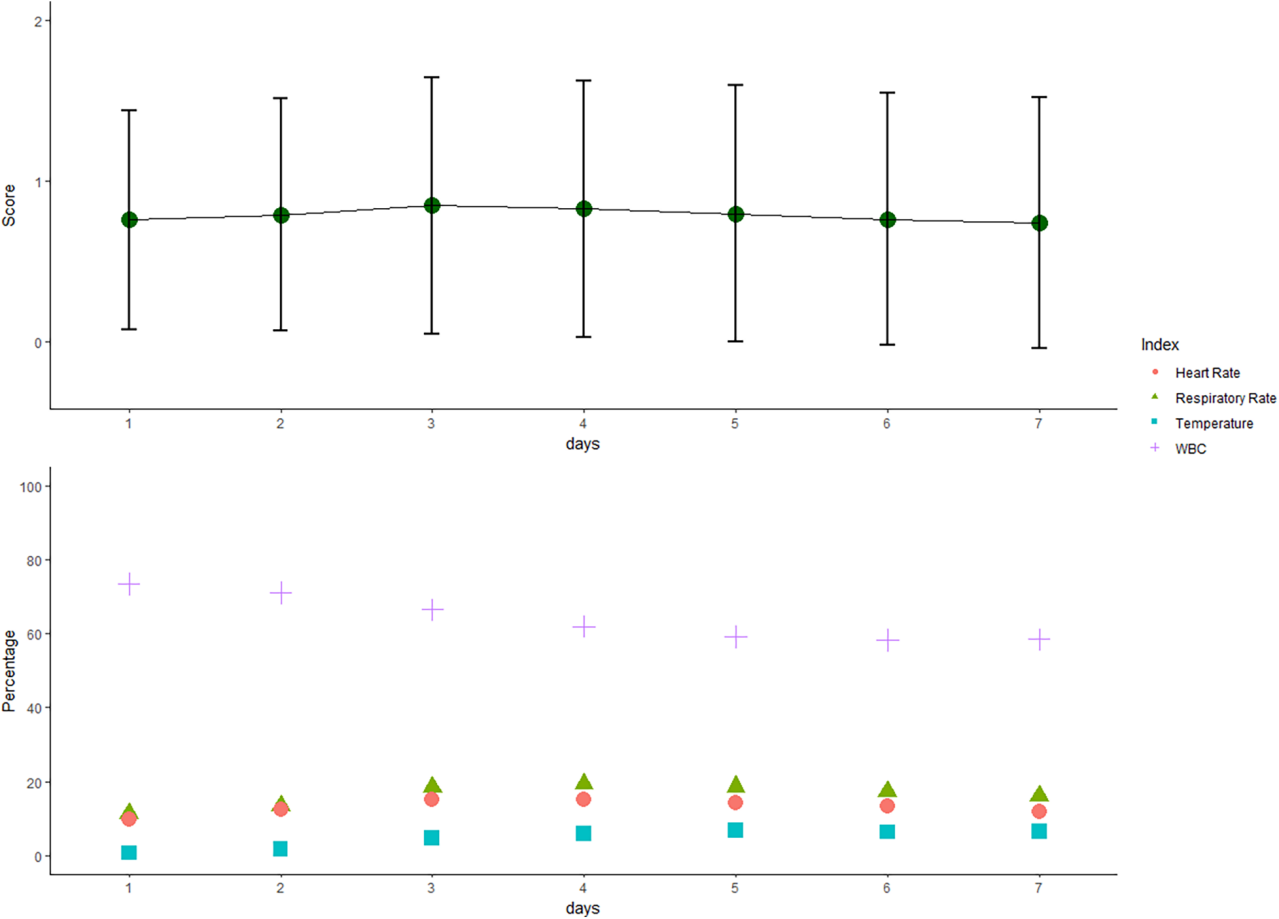
Systemic inflammatory response syndrome (SIRS) score over the first 7 days after craniotomy. The mean (sd of the mean) daily SIRS score increased over the first 3 days and decreased thereafter. Positive individual SIRS criteria (heart rate > 90 beats per minute, temperature > 38°C or <36°C, white blood cell (WBC) > 12,000/mm^3^ or <4,000/mm^3^, and respiratory rate > 20 breaths per minute) are presented as percentages in the 7 days after surgery.

The mean daily SIRS score increased over the first days and decreased thereafter (Figure 2). Abnormal WBC count contributed the most to the SIRS score followed by abnormalities in body temperature, respiratory rate, and heart rate. WBC count was abnormal in more than 50% of patient days. Although leukocytosis (>12,000 cells/mm^3^) more frequently occurred in the early phase, leukopenia (<4,000 cells/mm^3^) was more common in the delayed phase.

### SIRS positivity, early SIRS, and delayed SIRS

Over half of the patients (*n* = 6,725; 52.2%) developed SIRS within the first week after craniotomy. Early SIRS was diagnosed in 34.4% (4,181/12,887), delayed SIRS was diagnosed in 39.0% (5,026/12,887), and 47.8% of patients experienced early SIRS without delayed SIRS. While 2,544 patients (19.7%) developed SIRS from days 4 to 7 without having SIRS before day 3, 2,482 patients (19.3%) had continuous SIRS and 1,699 patients (13.2%) redeveloped SIRS.

### SIRS and 30-day mortality

The total 30-day mortality rate for our study population was 1.22% (*n* = 157). SIRS-positive patients had a mortality rate of 1.55% (*n* = 104) compared to 0.86% (*n* = 53) in SIRS-negative patients. Multivariate analysis showed that SIRS positivity was associated with higher 30-day mortality (OR, 1.57; 95% CI, 1.12–2.21; *p* = 0.009) (Supplementary Table S1). Importantly, delayed SIRS (adjusted OR, 1.53; 95% CI, 1.11–2.11; *p* = 0.01) was still associated with 30-day mortality, whereas early SIRS (adjusted OR, 1.27; 95% CI, 0.92–1.76; *p* = 0.15) was not associated with 30-day mortality (Table 2).

**Table 2:** Association of systemic inflammatory response syndrome (SIRS) with 30-day mortality in three separate models.

SIRS Variables	Univariable OR (95% CI)	*p*	Multivariate OR (95% CI)	*p*
SIRS	1.81 (1.30–2.52)	<0.001*	1.57 (1.12–2.21)	0.009*
Early SIRS	1.44 (1.04–1.98)	0.03*	1.27 (0.92–1.76)	0.15
Delayed SIRS	1.72 (1.26–2.36)	0.001*	1.53 (1.11–2.11)	0.01*

* Means statistically significance.

### Inflammatory biomarkers after craniotomy

**Figure 3:**
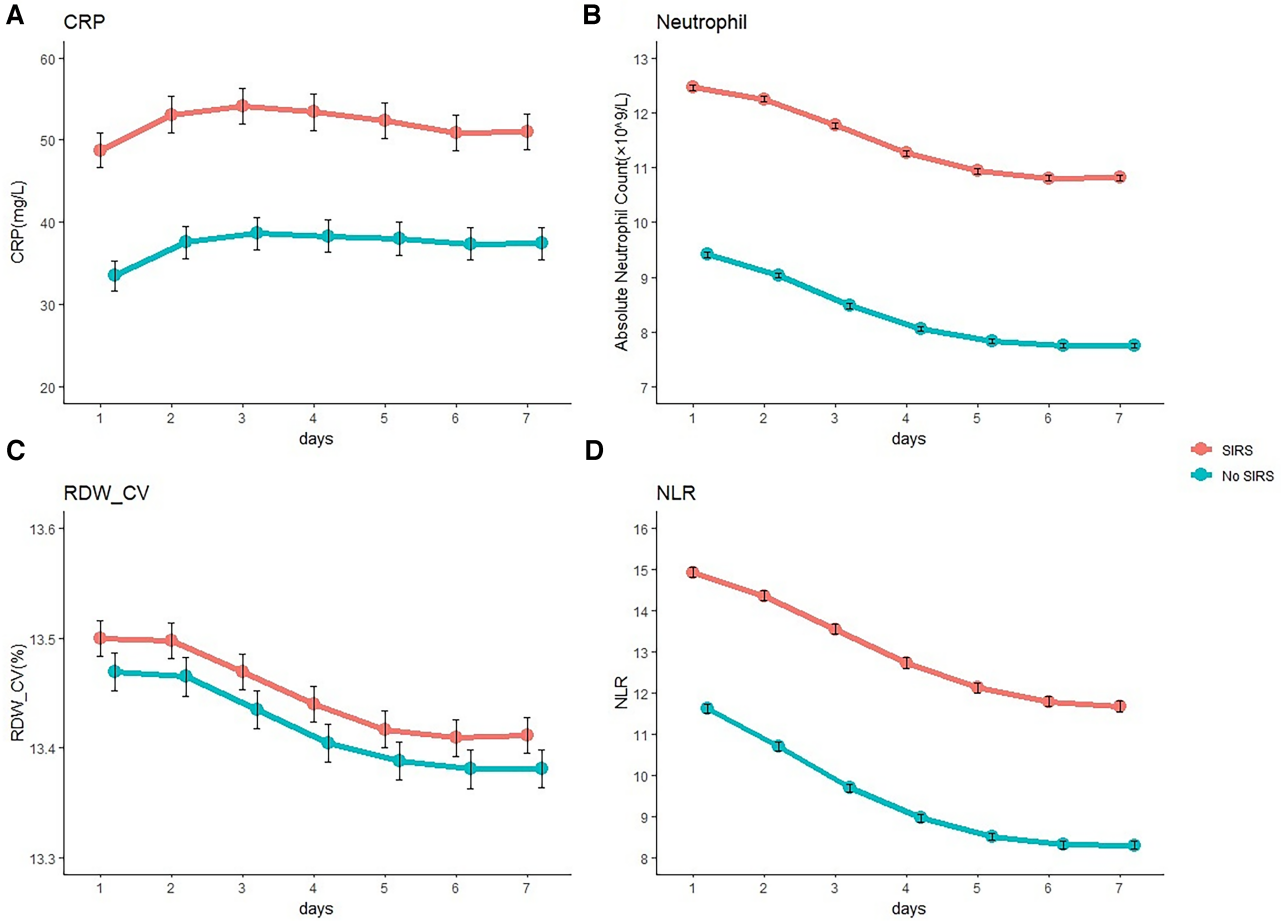
**Inflammatory biomarker level after craniotomy**. Mean C-reactive protein (CRP) (**A**), absolute neutrophil count (**B**), coefficient of variation of red blood cell distribution width (RDW- CV) (**C**), and neutrophil-lymphocyte ratio (NLR) (**D**) levels in patients after craniotomy according to the occurrence of systemic inflammatory response syndrome (SIRS).

The increase in inflammatory biomarkers after craniotomy is summarized in Figure 3. The CRP level of proinflammatory cytokines was increased in the first 3 days after craniotomy and subsequently decreased. SIRS-positive patients had higher CRP levels than those in SIRS-negative patients. Absolute neutrophil count, RDW-CV, and NLR subsequently decreased during the first week after surgery, and SIRS-positive patients also had a higher levels than those in SIRS-negative patients.

**Figure 4:**
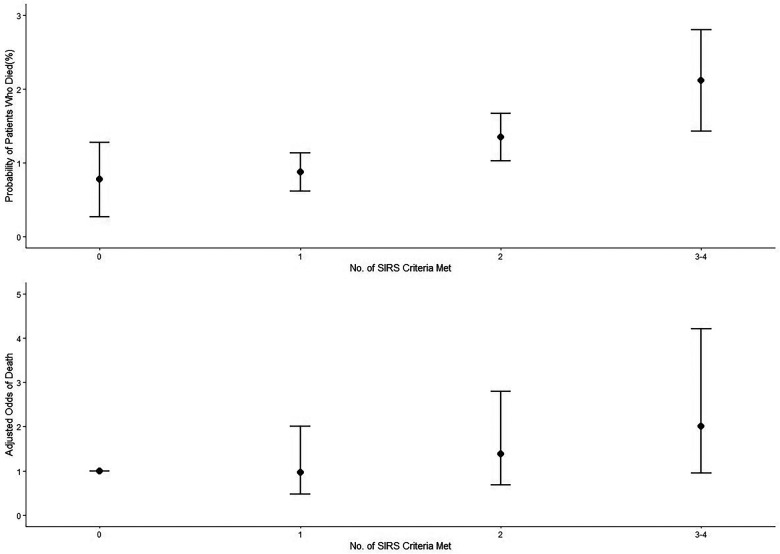
A 30-day mortality among patients after craniotomy, according to the number of SIRS criteria Met. SIRS criteria 3–4 were combined. SIRS, systemic inflammatory response syndrome.

In a model that included the worst SIRS score in the first 7 days after surgery from 0 to 4 and when we combined the 3–4 score (because of the small number of SIRS criteria 4), a 29% linear increase in 30-day mortality was associated with each additional SIRS criterion (odds ratio for each additional criterion, 1.29; 95% CI, 1.10–1.51; *p* = 0.002) (Figure 4).

**Figure 5:**
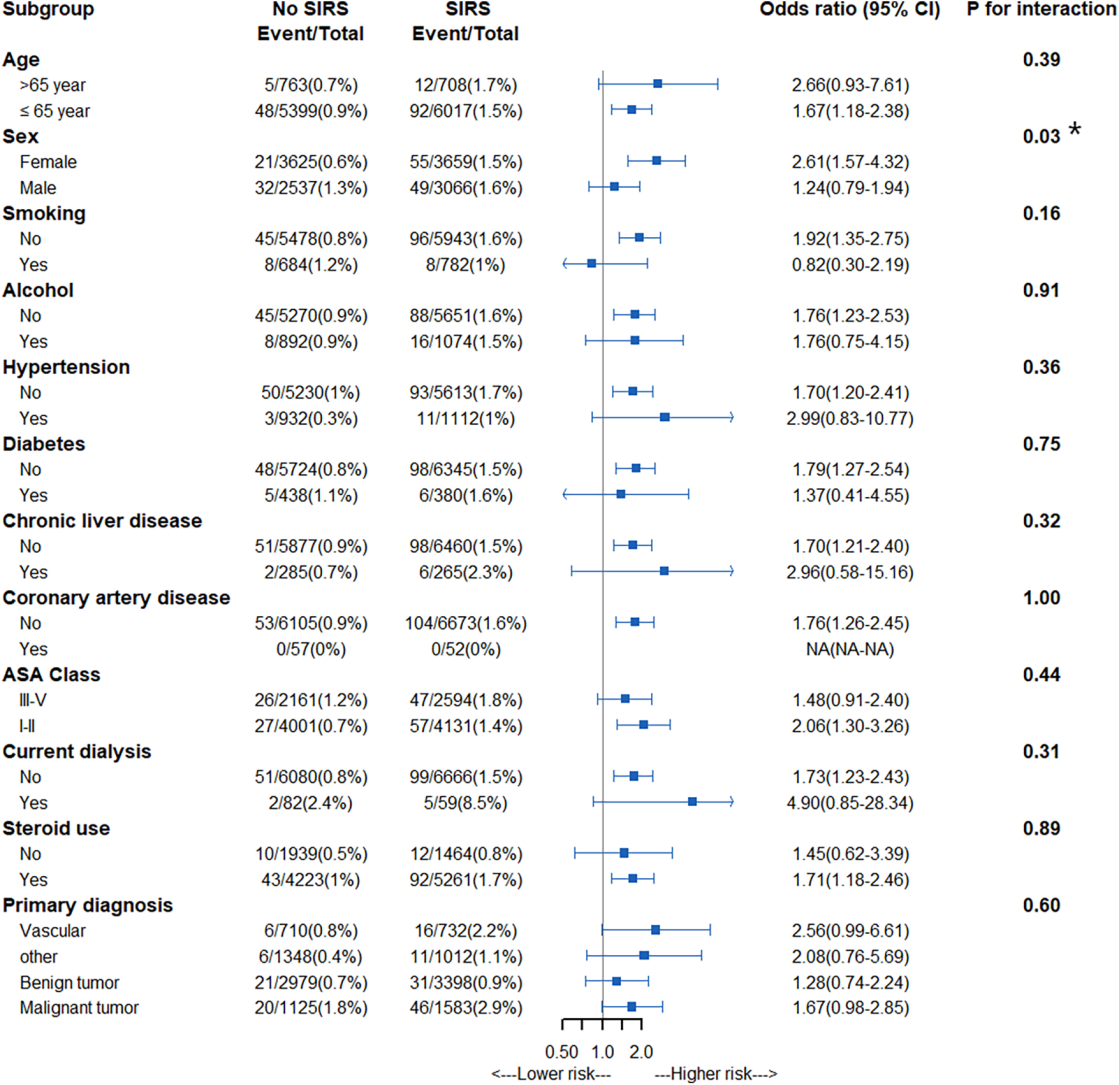
Subgroup analysis of adjusted association between SIRS positivity and 30-day mortality.

We further assessed interactions by variables on SIRS (Figure 5). There was no significant effect modification of the association between SIRS and mortality based on age, sex, alcohol abuse, current smoking, medical history of hypertension, diabetes, coronary artery disease, chronic liver disease, current dialysis, ASA class, perioperative steroid use, and primary diagnosis.

## Discussion

In this large cohort of 12,887 patients who underwent craniotomy without infection, postoperative SIRS was associated with increased 30-day postoperative mortality. This association was seen only in delayed SIRS, but not early SIRS. Moreover, abnormal WBC count contributed most to the SIRS score, followed by abnormalities in body temperature, respiratory rate, and heart rate.

To the best of our knowledge, this is the first study of postoperative SIRS in patients who underwent craniotomy. Previous studies (18–24) have evaluated postoperative SIRS in patients after various types of surgeries and showed that postoperative SIRS was associated with poor outcomes. Moreover, our research sample was larger and focused on noninfectious SIRS, which resulted in a higher predictive value for the perioperative outcome.

In our study, postoperative 30-day mortality was associated with delayed SIRS, but not early SIRS. This finding was consistent with Rass's research (5) and suggested a potential contribution of surgical stress, specifically the neurogenic mechanism, to the development of early SIRS, whereas systemic inflammation may largely trigger the evolution of delayed SIRS. Sympathetic stress responses mediated by the release of catecholamines and cortisol are common after craniotomy, leading to increased heart rate, blood pressure, and upregulation of proinflammatory cytokines and may persist for several days potentially contributing to early and delayed SIRS. Delayed SIRS may also be caused by infections that have not been isolated or documented. From a clinical standpoint, continuous monitoring of delayed SIRS plays a significant role in providing crucial clinical guidance. Once delayed SIRS is identified, it is crucial to actively monitor the stress response of patients and investigate potential infections, intensifying surveillance, as this has the potential to improve the prognosis of patients.

When analyzing the predictive value of each SIRS criterion, we found that every criterion was independently associated with postoperative 30-day mortality. Abnormal WBC had the highest proportional contribution to the SIRS score. Leukocytosis after surgery is a complex phenomenon with multiple underlying mechanisms, including immune activation due to tissue damage or infection, stress-induced hormonal responses, and changes in leukocyte mobilization from the bone marrow to the circulation. Further studies are required to fully understand the pathways underlying postoperative leukocytosis and develop effective treatments to reduce SIRS-related complications (3).

The mechanism of postoperative SIRS after craniotomy is unclear, and some theories may contribute to this. First, surgery itself can cause significant tissue damage, resulting in the release of damage-associated molecular patterns and pathogen-associated molecular patterns. These molecules can activate the innate immune system, leading to the production of proinflammatory cytokines and chemokines. Second, the stress response can activate the hypothalamic–pituitary–adrenal axis, leading to the release of cortisol and other stress hormones that can modulate the immune response. Third, recent studies suggest that the gut–brain axis plays a crucial role in the development of postoperative SIRS after craniotomy. Disruption of the gut microbiome due to antibiotic prophylaxis and anesthesia may contribute to SIRS development. The gut microbiota interacts with the immune system, modulating its function and contributing to the development of inflammation.

This study had a number of strengths and was based on one of the largest datasets, with a cohort of 12,887 adult patients undergoing craniotomy in a tertiary hospital, covering a wide geographical area. Moreover, we used a high-quality database to obtain death and obtained the continuous variables after surgery.

There are several limitations to our study. First, this was a single-center study in China. Therefore, the results may not be generalizable to other settings. Second, there is no gold standard for determining infections, and we obtained data according to discharge diagnosis. Thus, we might have included patients who were infected and excluded others who were not. Third, we only studied four inflammatory biomarkers because others were not available. Fourth, in patients undergoing craniotomy, multiple potential causes of SIRS may coexist, making it difficult or even impossible to pinpoint the precise cause.

## Conclusions

Postoperative SIRS commonly occurs after craniotomy and is associated with 30-day postoperative mortality. This association was observed only in patients with delayed SIRS but not early SIRS. Moreover, abnormal WBC counts contributed the most to the SIRS score.

## Data Availability

The raw data supporting the conclusions of this article will be made available by the authors, without undue reservation.

## References

[1] Malham GM Souter MJ. Systemic inflammatory response syndrome and acute neurological disease. Br J Neurosurg. (2001) 15(5):381–7. 10.1080/02688690120082378 11708540

[2] Menger MD Vollmar B. Surgical trauma: hyperinflammation versus immunosuppression? Langenbecks Arch Surg. (2004) 389(6):475–84. 10.1007/s00423-004-0472-0 15173946

[3] Margraf A Ludwig N Zarbock A Rossaint J. Systemic inflammatory response syndrome after surgery: mechanisms and protection. Anesth Analg. (2020) 131(6):1693–707. 10.1213/ane.0000000000005175 33186158

[4] Mokart D Merlin M Sannini A Brun JP Delpero JR Houvenaeghel G Procalcitonin, interleukin 6 and systemic inflammatory response syndrome (SIRS): early markers of postoperative sepsis after major surgery. Br J Anaesth. (2005) 94(6):767–73. 10.1093/bja/aei143 15849208

[5] Rass V Gaasch M Kofler M Schiefecker AJ Ianosi BA Rhomberg P Systemic inflammatory response syndrome as predictor of poor outcome in nontraumatic subarachnoid hemorrhage patients. Crit Care Med. (2018) 46(12):e1152–9. 10.1097/ccm.0000000000003429 30252711

[6] Jacome T Tatum D. Systemic inflammatory response syndrome (SIRS) score independently predicts poor outcome in isolated traumatic brain injury. Neurocrit Care. (2018) 28(1):110–6. 10.1007/s12028-017-0410-y 28547319

[7] Boehme AK Comeau ME Langefeld CD Lord A Moomaw CJ Osborne J Systemic inflammatory response syndrome, infection, and outcome in intracerebral hemorrhage. Neurol Neuroimmunol Neuroinflamm. (2018) 5(2):e428. 10.1212/nxi.0000000000000428 29318180 PMC5745360

[8] Wessell AP Kole MJ Cannarsa G Oliver J Jindal G Miller T A sustained systemic inflammatory response syndrome is associated with shunt-dependent hydrocephalus after aneurysmal subarachnoid hemorrhage. J Neurosurg. (2019) 130(6):1984–91. 10.3171/2018.1.JNS172925 29957109

[9] Takenaka K Ogawa E Wada H Hirata T. Systemic inflammatory response syndrome and surgical stress in thoracic surgery. J Crit Care. (2006) 21(1):48–53. 10.1016/j.jcrc.2005.07.001 16616623

[10] Fink-Neuboeck N Lindenmann J Bajric S Maier A Riedl R Weinberg AM Clinical impact of interleukin 6 as a predictive biomarker in the early diagnosis of postoperative systemic inflammatory response syndrome after major thoracic surgery: a prospective clinical trial. Surgery. (2016) 160(2):443–53. 10.1016/j.surg.2016.04.004 27206334

[11] MacCallum NS Finney SJ Gordon SE Quinlan GJ Evans TW. Modified criteria for the systemic inflammatory response syndrome improves their utility following cardiac surgery. Chest. (2014) 145(6):1197–203. 10.1378/chest.13-1023 24576975

[12] Hirai S Hamanaka Y Mitsui N Isaka M Sutoh M. Prospective study of systemic inflammatory response syndrome after cardiac surgery as a effective indicator. Kyobu Geka. (2004) 57(6):455–8. 15202264

[13] Haga Y Beppu T Doi K Nozawa F Mugita N Ikei S Systemic inflammatory response syndrome and organ dysfunction following gastrointestinal surgery. Crit Care Med. (1997) 25(12):1994–2000. 10.1097/00003246-199712000-00016 9403749

[14] Lahiri R Derwa Y Bashir Z Giles E Torrance HD Owen HC Systemic inflammatory response syndrome after major abdominal surgery predicted by early upregulation of TLR4 and TLR5. Ann Surg. (2016) 263(5):1028–37. 10.1097/sla.0000000000001248 26020106

[15] Iwasaki A Shirakusa T Maekawa T Enatsu S Maekawa S. Clinical evaluation of systemic inflammatory response syndrome (SIRS) in advanced lung cancer (T3 and T4) with surgical resection. Eur J Cardiothorac Surg. (2005) 27(1):14–8. 10.1016/j.ejcts.2004.09.006 15621464

[16] Chan KW. The Chinese hukou system at 50. Eurasian Geogr Econ. (2009) 50(2):197–221. 10.2747/1539-7216.50.2.197

[17] American College of Chest Physicians/Society of Critical Care Medicine Consensus Conference: definitions for sepsis and organ failure and guidelines for the use of innovative therapies in sepsis. Crit Care Med. (1992) 20(6):864–74. 10.1097/00003246-199206000-00025 1597042

[18] Chawla BK Teitelbaum DH. Profound systemic inflammatory response syndrome following non-emergent intestinal surgery in children. J Pediatr Surg. (2013) 48(9):1936–40. 10.1016/j.jpedsurg.2013.05.065 24074671 PMC3787315

[19] Ikeda H. Specificity of systemic inflammatory response syndrome during the peri-operative period in patients with GH-secreting adenoma. Cytokine. (2009) 46(1):92–5. 10.1016/j.cyto.2008.12.026 19264503

[20] Gago R Vilá S Vélez-Rivera J Vilá LM. Severe systemic inflammatory response syndrome immediately after spinal surgery in a patient with axial gout. BMJ Case Rep. (2018) 2018:bcr2017222474. 10.1136/bcr-2017-222474 29367221 PMC5786998

[21] Dieleman JM Peelen LM Coulson TG Tran L Reid CM Smith JA Age and other perioperative risk factors for postoperative systemic inflammatory response syndrome after cardiac surgery. Br J Anaesth. (2017) 119(4):637–44. 10.1093/bja/aex239 29121297

[22] Chaikittisilpa N Krishnamoorthy V Lele AV Qiu Q Vavilala MS. Characterizing the relationship between systemic inflammatory response syndrome and early cardiac dysfunction in traumatic brain injury. J Neurosci Res. (2018) 96(4):661–70. 10.1002/jnr.24100 28573763 PMC5712282

[23] Boeken U Feindt P Micek M Petzold T Schulte HD Gams E. Procalcitonin (PCT) in cardiac surgery: diagnostic value in systemic inflammatory response syndrome (SIRS), sepsis and after heart transplantation (HTX). Cardiovasc Surg. (2000) 8(7):550–4. 10.1016/s0967-2109(00)00070-3 11068216

[24] Stoppelkamp S Veseli K Stang K Schlensak C Wendel HP Walker T. Identification of predictive early biomarkers for sterile-SIRS after cardiovascular surgery. PLoS One. (2015) 10(8):e0135527. 10.1371/journal.pone.0135527 26263001 PMC4532358

