# Supplementary material for: Postoperative systemic inflammatory response syndrome predicts increased mortality in patients after elective craniotomy
Source: Front Surg. 2024 Jan 4;10:1331073. doi: 10.3389/fsurg.2023.1331073 (PMC10794578; doi:10.3389/fsurg.2023.1331073)
Supplement: Supplementary file 1 [file Table1.docx]

**Table S1 Factors Associated with Mortality at 30 Days**

| Characteristics | Univariable | | Multivariable | |
| --- | --- | --- | --- | --- |
|  | OR (95% CI) | P | OR (95% CI) | P |
| Demographics | | | | |
| Age>65 years | 0.98(0.97- 0.99) | 0.005 | 0.99(0.98- 1.00) | 0.075 |
| Female | 0.72 (0.52- 0.98) | 0.04 | 0.85(0.62-1.17) | 0.328 |
| Smoking | 0.88(0.52- 1.48) | 0.638 | NA | NA |
| Alcohol | 1.00(0.65- 1.55) | 0.991 | NA | NA |
| Medical history | | | | |
| Hypertension | 0.52(0.30- 0.90) | 0.019 | 0.57(0.32-1.03) | 0.061 |
| Diabetes | 1.11 (0.60- 2.06) | 0.733 | NA | NA |
| Chronic liver disease | 1.21 (0.59- 2.47) | 0.606 | NA | NA |
| Current dialysis | 4.39(2.02-9.54) | <0.001 | 5.23 (2.35-11.64) | <0.001 |
| Coronary artery disease | 0.00(0.00-Inf) | 0.972 | NA | NA |
| Primary diagnosis | | | | |
| Benign | 1 [Reference] |  | 1 [Reference] |  |
| Malignant | 3.04(2.11- 4.38) | <0.001 | 2.78(1.90- 4.06) | <0.001 |
| Other | 0.88(0.51- 1.53) | 0.656 | 1.24(0.70- 2.21) | 0.458 |
| Vascular | 1.88(1.14- 3.11) | 0.013 | 2.49(1.48- 4.20) | 0.001 |
| ASA class | | | | |
| I-II | 1 [Reference] |  | 1 [Reference] |  |
| III-V | 1.49(1.09- 2.05) | 0.013 | 1.57(1.13-2.19) | 0.007 |
| Steroid use | 2.22(1.41- 3.49) | 0.001 | 1.71(1.06-2.78) | 0.029 |
| Surgery time | 1.21(1.13- 1.28) | <0.001 | 1.15(1.07-1.25) | <0.001 |
| Intraoperative blood loss | 1.00(1.00- 1.00) | <0.001 | 1.00(1.00-1.00) | 0.001 |
| SIRS | 1.81(1.30- 2.52) | <0.001 | 1.57(1.12-2.21) | 0.009 |

ASA: American Society of Anesthesiologists; NA: not available

C-statistics: 0.728
